# 

**Published:** 1884-08-20

**Authors:** 


### TABLE OF CONTENTS.

ORIGINAL AND SELECTED ARTICLES:

Interesting Case of Skull Injury, by Wm. Perrin Nicolson, M.D., Professor of Anatomy in Southern Medical College, Atlanta, Ga., and Surgeon to Ivy Street Hospital, 281. Gossypium Herbaceum—Some Remarks on its Parturient Properties, by W. C. Bellamy, M.D, Atlanta, Ga., 283. Obstetrical Problems, 285. Puerperal Eclampsia, 288. The Neutral Treatment in Fevers, 290. Large Doses of Calomel in True Croup, 293. Functions of the Spleen, 295. Pediatric Aphorisms, 296. Anæsthetics in Cataract Operations, 298.

ABSTRACTS AND GLEANINGS:

Advice to the Medical Witness, 299. Chloroform as an Anti-Ferment, 300. Traumaticin as a Medium for the Application of Certain Substances in Diseases of the Skin; Opacities of the Cornea, 301 Painful Ulcers of the Cornea; Strychnia in Delirium Tremens; To Prevent Necessity for Inducing Abortion, 302. Rapid Absorption of Pleuritic Effusion by use of Salt and Cutting-off Liquids, 303. Enemas in Severe forms of Constipation; Nasal Hemorrhage—Plugging the Nose; To Abort a Stye, 304. Case of Uterine Disease; The Poison of the Skunk, 305. Nurses' Sore Mouth; Boils; Liquor Hydrargyri Bichloridi in Dysentery, 306. Sorghum and Typho-Malarial Fever; Paraldehyde as a Hypnotic, 307. Injections of Glycerine and Carbolic Acid for the Preservation of Bodies for Dissection; Warts, 308. Escharotics; Nævus—Local Application of Liquor Arsenicalis; Benzoate of Soda in Infantile Diarrhœa, 309. Vanillism; Disinfectants; Consumption from Street Dust, 310.

SCIENTIFIC ITEMS:

The Frontiers of Madness; Paraffine Paper, 311. Loganin; Dentistry in the United States; Water-glass Polish for Floors; Dilute Solution of Corrosive Sublimate; Bringing up Children, 312.

PRACTICAL NOTES AND FORMULÆ.

The Administration of Cotoine, 313. No. 3, Elixir Ammonii Valerianatis; Tartaric Acid in Diphtheria; Loomis' Tonic Bitters; Walnut Hair Dye, 314. Diarrhœa and Dysentery of Children; For Diarrhœa and Dysentery; Dysmenorrhœa; Diarrhœa in Children, 315.

EDITORIALS AND MISCELLANEOUS.

Editorial Notices, 316; The Ivy Street Hospital; American Public Health Association, 317. Criminal Abortion; Books and Pamphlets Received, 318. Important Notice, 319. Receipted; Special Notices, 320.

